# Enhanced effector function of cytotoxic cells in the induced sputum of COPD patients

**DOI:** 10.1186/1465-9921-11-76

**Published:** 2010-06-11

**Authors:** Richard A Urbanowicz, Jonathan R Lamb, Ian Todd, Jonathan M Corne, Lucy C Fairclough

**Affiliations:** 1 COPD Research Group, Nottingham Respiratory Biomedical Research Unit, The University of Nottingham, NG7 2UH, UK; 2 Immunology and Infection Section, Division of Cell and Molecular Biology, Faculty of Natural Sciences, Imperial College London, South Kensington, London, SW7 9AZ, UK; 3 Department of Respiratory Medicine, Nottingham University Hospitals, NG7 2UH, UK

## Abstract

**Background:**

We have previously shown that NK (CD56^+^CD3^-^) and NKT-like (CD56^+^CD3^+^) cells are reduced in both numbers and cytotoxicity in peripheral blood. The aim of the present study was to investigate their numbers and function within induced sputum.

**Methods:**

Induced sputum cell numbers and intracellular granzyme B and perforin were analysed by flow cytometry. Immunomagnetically selected CD56^+ ^cells (NK and NKT-like cells) were used in an LDH release assay to determine cytotoxicity.

**Results:**

The proportion of NK cells and NKT-like cells in smokers with COPD (COPD subjects) was significantly higher (12.7% and 3%, respectively) than in healthy smokers (smokers) (5.7%, p < 0.01; 1%, p < 0.001) and non-smoking healthy subjects (HNS) (4.2%, p < 0.001; 0.8%, p < 0.01). The proportions of NK cells and NKT-like cells expressing *both *perforin *and *granzyme B were also significantly higher in COPD subjects compared to smokers and HNS. CD56^+ ^cells from COPD subjects were significantly more cytotoxic (1414 biological lytic activity) than those from smokers (142.5; p < 0.01) and HNS (3.8; p < 0.001) and were inversely correlated to FEV_1_. (r = -0.75; p = 0.0098).

**Conclusion:**

We have shown an increased proportion of NK and NKT-like cells in the induced sputum of COPD subjects and have demonstrated that these cells are significantly more cytotoxic in COPD subjects than smokers and HNS.

## Background

Chronic obstructive pulmonary disease (COPD) is a complex condition consisting of emphysema, respiratory bronchiolitis and chronic bronchitis [1]. It is projected to be the fifth commonest cause of morbidity and the third leading cause of death worldwide by 2030 [2]. Tobacco smoking is established as the main aetiological factor for COPD and it is now accepted that COPD is an inflammatory disorder.

Inflammation of the airways is present in COPD with increased numbers of inflammatory cells from both the innate and adaptive host response, such as macrophages and lymphocytes in the airway wall [3] and neutrophils in the airway lumen [4]. Many of these cells have the potential to cause the damage seen in the airways of patients with COPD, including three main heterogeneous and functionally distinct classes of human killer cells; namely CD8^+ ^T lymphocytes, CD56^+^CD3^- ^(natural killer; NK) cells and CD56^+^CD3^+ ^(NKT-like) cells [5].

Killer cells lyze their target cells by two mechanisms: membranolysis, in which secreted molecules, such as perforin and granzymes, form pores in the membrane of target cells [6]; and apoptosis, mediated through the triggering of apoptosis-inducing (Fas-like) surface molecules of the target cell [7].

Airway inflammation has been studied in bronchial biopsy samples and bronchoalveolar lavage (BAL) fluid [8]. Recently, it has been reported that soluble granzyme B levels and the proportion of T cells expressing intracellular granzyme B or perforin were increased in the BAL of both current and ex-smokers smokers with COPD [9]. Others have shown a decrease in NK cell numbers in the BAL of patients with chronic bronchitis [10], but to date, no in-depth study of BAL NKT-like cells in patients with COPD has been performed.

Studies in sputum have demonstrated increased perforin expression and cytotoxic activity of CD8^+ ^lymphocytes [11], although the measurement may have included two other types of killer cells (namely NK and NKT-like cells) as these can also express CD8 on the cell surface. Information on the functional phenotypes of NK and NKT-like cells in induced sputum in COPD is limited.

We have previously shown that there are significant differences in the proportions, subsets, intracellular proteins and cytotoxic activities of NK cells and NKT-like cells in the peripheral blood of COPD subjects. Specifically, peripheral cell numbers and cytotoxicity of these cells were reduced in COPD [12]. However a study of peripheral cells does not necessarily reflect changes in the airway. The first step of assessing the potential importance of these cells in the inflammatory process would be to assess their numbers and function within the airways. Therefore, in this study we have extended the findings of our previous study to investigate, within induced sputum, the number and cytotoxic function of the three main classes of human killer cells; CD8^+ ^T lymphocytes, NK cells and NKT-like cells.

## Methods

### Study population and procedures

The Nottingham Local Research Ethics Committee approved the study protocol and written informed consent was obtained from the 26 subjects before entering the blinded study. Of these, the 11 participants diagnosed as having COPD (COPD subjects), according to the ATS guidelines, were current or ex-smokers who had accrued at least a 20 pack year smoking history and had an FEV_1 _below 80% of predicted with an FEV_1_/FVC ratio of <70% and reversibility to an inhaled beta-2 agonist of <10% or <200 mls absolute improvement. Ten healthy smokers (smokers), defined as smokers without airflow limitation, and five healthy non-smokers (HNS), with an FEV_1 _above 80% of predicted, were recruited and matched for age and for the healthy smokers, smoking history, as closely as possible. Table 1 details the demographic and spirometric data of the subjects. Participants were excluded if they had a history of tuberculosis (TB), a clinical suspicion of current infection, history of physician diagnosed asthma or a positive skin prick test response to grass pollen, house dust mite, cat dander and dog hair (ALK-Abelló). COPD subjects were also excluded if they had had an exacerbation within the previous 6 weeks, were α1-anti-trypsin deficient or had other lung disease.

**Table 1:** Demographic and spirometric values of the studied groups

	HNS	Smokers	COPD subjects
**Subjects**	5	10	11
**Age (years)**	51 (42-68)	51 (44-68)	63 (44-73)
**Gender (M/F)**	1/4	5/5	6/5
**Packs/yrs**	0 (0)	35 (19-51)	51 (27-77)
**Smoking status (Current/Ex)**	0	10/0	5/6
**Chronic bronchitis** **(Yes/No)**	0	4/6	7/4
**FEV_1 _(% pred)**	112 (88-124)	100 (89-122)	59 (37-73)
**FEV_1_/FVC (%)**	78 (72-86)	74 (73-82)	54 (39-69)
**ΔFEV_1 _post bronch**	2.2 (1.1-3.5)	1.6 (1.1-2.7)	3.9 (3.6-4.3)
**BMI (kg/m^2^)**	26.4 (18.9-29.3)	23.8 (20.0-31.0)	24.7 (19.3-34.0)
**Inhaled GCS (on/off)**	N/A	N/A	7/4
**MRC dyspnoea scale**	N/A	N/A	3 (2-4)
**Distance walked in 6 min (m)**	N/A	N/A	291 (168-554)
**BODE Index**	N/A	N/A	5 (1-7)

Results are expressed as median with range in brackets.

HNS, non-smoking healthy subjects; COPD, chronic obstructive pulmonary disease; FEV_1_, forced expiratory volume in 1 second; pred, predicted value; FVC, forced vital capacity; GCS, Glucocorticosteroids.

### Sputum induction

Sputum was induced by inhalation of hypertonic saline as described previously [13] with the difference that the subject was given salbutamol (400 mg) via a volumatic, rather than 1 mg terbutaline. The sample was placed on ice and processed within 1 hour.

### Cell isolation and fractionation

The sputum was concentrated in a petri dish on ice, weight was calculated and 0.1% dithiothreitol (DTT) was added at a 4× weight/volume ratio. Sputum was dispersed, vortexed and then placed on a roller mixer. An equal volume of PBS was then added, vortexed and filtered through 48 μm nylon gauze. An aliquot for cell counting to assess viability and squamous cell contamination was taken. The remaining sample was centrifuged and resuspended.

CD56^+ ^cells were isolated using α-CD56 microbeads (Miltenyi Biotech Ltd) according to manufacturers' instructions. Briefly, cells were incubated for 15 minutes at 4°C with α-CD56 microbeads and separated on a refrigerated MS column using cold PBS containing 1% FCS and 0.4% EDTA. The resulting positive fraction was washed and resuspended in RPMI 1640 medium. Following isolation, all fractions were washed, counted and purity confirmed at ≥90% by flow cytometric analysis.

### Flow cytometric analysis

Cells were washed with PBA, fixed in 3% formaldehyde in isotonic azide free solution and given a final wash with PBA - 0.04% saponin with 10% FCS. Labelled antibodies (Table 2) were added at the recommended concentration and the cells were incubated for two hours at 4°C in the dark. Excess antibody was removed by washing and cells were stored in 0.5% formaldehyde in isotonic azide free solution at 4°C. Flow cytometric analysis of these antibody labelled cells was performed using an EPICS Altra (Beckman Coulter). Fifty thousand live-gated events were collected for each sample and isotype matched antibodies were used to determine binding specificity. Data were analysed using WEASEL version 2.3 (WEHI). Necrotic cells were excluded from analysis according to their forward and side scatter characteristics.

**Table 2:** Antibodies used for flow cytometry

Antigen	Fluorochrome	Isotype	Clone	Source
CD3	ECD PC7	Mouse IgG1	UCHT1	Beckman Coulter, Luton, UK
CD4	FITC PC5	Mouse IgG1	13B8.2	Beckman Coulter, Luton, UK
CD8	PC5 APC	Mouse IgG1	B9.11	Beckman Coulter, Luton, UK
CD8	ECD	Mouse IgG1	SFCl21Thy2D3	Beckman Coulter, Luton, UK
CD16	PC7	Mouse IgG1	3G8	Beckman Coulter, Luton, UK
CD19	PC5	Mouse IgG1, k	J4.119	Beckman Coulter, Luton, UK
CD45RA	FITC PE	Mouse IgG1	ALB11	Beckman Coulter, Luton, UK
CD45RO	ECD	Mouse IgG2a	UCHL1	Beckman Coulter, Luton, UK
CD56	PE PC5 PC7	Mouse IgG1	N901	Beckman Coulter, Luton, UK
CD62L	PC5	Mouse IgG1	DREG56	Beckman Coulter, Luton, UK
Granzyme B	FITC	Mouse IgG1k	GB11	Becton Dickinson, Oxford, UK
Perforin	PE	Mouse IgG2b	(G9	Becton Dickinson, Oxford, UK
CXCR3	PE	Mouse IgG1, k	1C6/CXCR3	Becton Dickinson, Oxford, UK
VLA-4	FITC	Mouse IgG1	HP2/1	Beckman Coulter, Luton, UK

ECD, phycoerythrin-Texas Red-x; FITC, fluorescein isothiocyanate; PC5, phycoerythrin-cyanin 5.1; PC7, phycoerythrin-cyanin 7; PE, phycoerythrin

### Cytotoxicity assay

A commercially available lactate dehydrogenase (LDH) kit (CytoTox 96 Non-Radioactive Cytotoxicity Assay, Promega) was used with erythroleukaemic K562 cells (ECACC) as the target cell line. Briefly, the effector and target cells were mixed at a ratio of 5:1, plated in quadruplicate on a 96-well U-bottomed plate and incubated for 4 h at 37°C in a humidified atmosphere containing 5% CO_2_. After incubation, the plate was centrifuged, the supernatants collected and incubated for 30 min at room temperature with the Substrate Mix provided with the kit to detect LDH activity. A stop solution was added and the absorbance of the sample was measured at 490 nm on an Emax precision microplate reader (Molecular Devices) using SOFTmax software (Molecular Devices). The amount of cell-mediated cytotoxicity was calculated by subtracting the spontaneous LDH released from the target and effector cells from the LDH released by lysed target cells, using the following equation:

Specific lysis (%) = {(effector/target cell mix - spontaneous effector LDH release - spontaneous target LDH release)/(maximum target LDH release - spontaneous target LDH release)}x100

The biological lytic ability was then calculated by multiplying the lytic activity per cell by the total number of CD56^+ ^cells per gram of induced sputum.

### Statistical analysis

The statistical analysis was performed with Prism software, version 4.0c (GraphPad). Normality was detected using the Kolmogorov-Smirnov test. As some data were non-normally distributed all are expressed as median (range), unless otherwise stated. Differences between the three groups of subjects were tested using the non-parametric Kruskal-Wallis test with *post hoc *pairwise comparisons made by the Dunn's Multiple Comparison test to determine which pair was statistically significantly different. P values of less than 0.05 were considered to indicate statistical significance.

## Results

There was no statistical difference between groups in terms of age (Kruskal-Wallis test). Furthermore, there was no significant difference in smoking history between COPD subjects and healthy smokers.

### Comparison of constituent cells in induced sputum

The viability of the cells (% total) in induced sputum did not differ between COPD subjects, smokers and HNS (median, range): 83 (75-94), 82 (72-93) and 86 (75-94), respectively. In all samples, more than 90% of the cells were non-squamous cells. The total cell count (TCC; g^-1^) was significantly higher in COPD subjects than in smokers and HNS (Table 3). The TCC was also higher in smokers than in HNS (Table 3). The differences in cellular parameters amongst the three groups are presented in Table 3. The proportions of neutrophils and lymphocytes were higher in COPD subjects than in the other two groups, with the proportion of macrophages lower.

**Table 3:** Cellular populations in sputum of HNS, smokers and COPD subjects (median, range)

	HNS (group A, n = 5)	Smokers (group B, n = 10)	COPD Subjects (group C, n = 11)	p values
TCC, × 10^7 ^cells/g	1.24 (0.4-1.9)	4.93 (2-7.4)	10.66 (5.2-14.9)	COPD vs Smokers p < 0.05 COPD vs HNS p < 0.001

Neutrophils, × 10^7 ^cells/g	0.53 (0.14-0.63)	2.37 (1.23-4.06)	7.94 (3.82-12.13)	COPD vs Smokers p < 0.01 COPD vs HNS p < 0.001

Macrophages, × 10^7 ^cells/g	0.58 (0.21-0.95)	2.41 (0.88-3.99))	1.89 (0.76-2.75)	COPD vs HNS p < 0.05 Smokers vs HNS p < 0.01

Lymphocytes, × 10^7 ^cells/g	0.01 (0.00-0.02)	0.16 (0.04-0.51)	0.90 (0.28-1.44)	COPD vs Smokers p < 0.05 COPD vs HNS p < 0.001

Eosinophils, × 10^7 ^cells/g	0.009 (0.001-0.023)	0.022 (0.002-0.056)	0.122 (0.019-0.203)	NS

				

Neutrophils, %	35.3 (29.8-41.9)	55.5 (38.1-67.2)	74.2 (59.4-82.7)	COPD vs Smokers p < 0.01 COPD vs HNS p < 0.001

Macrophages, %	51.4 (40.9-63.6)	42.8 (35.5-62.4)	19.6 (6.8-27.2)	COPD vs Smokers p < 0.001 COPD vs HNS p < 0.01

Lymphocytes, %	0.9 (0.3-1.3)	3.9 (0.7-7.4)	9.5 (3.2-13.5)	COPD vs Smokers p < 0.05 COPD vs HNS p < 0.001

Eosinophils, %	0.7 (0-1.2)	0.6 (0.1-0.9)	1.3 (0.2-1.9)	NS

Results are expressed as median of total non-squamous cells with range in brackets

**Figure 1:**
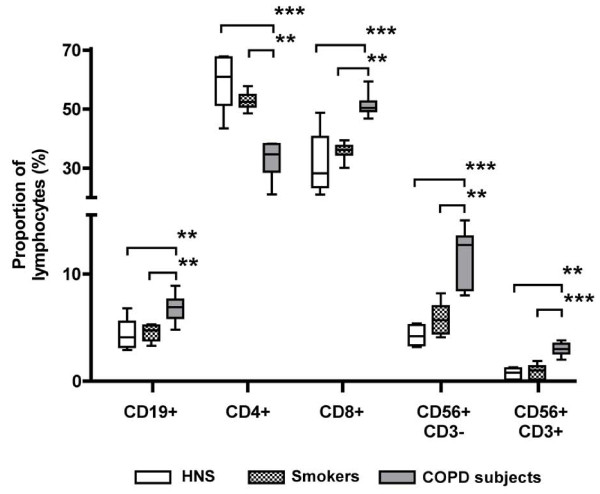
**Proportion and type of lymphocytes from the induced sputum of HNS (n = 5), smokers (n = 10) and COPD subjects (n = 11)**. Results show a significant increase in the proportion of all three cytotoxic cells (CD8^+^, NK and NKT-like cells) in COPD subjects compared to HNS and smokers. Cell types were determined by flow cytometric analysis of monoclonal antibodies. CD19, B cells; CD4, T helper cells; CD8, cytotoxic killer cells; CD56^+^CD3^-^, NK cells; CD56^+^CD3^+^, NKT-like cells. **: p < 0.01, ***: p < 0.001.

Flow cytometric analysis of the lymphocytes showed that the proportions of CD8^+ ^T lymphocytes, NK (CD56^+^CD3^-^) cells and NKT-like (CD56^+^CD3^+^) cells were significantly higher in COPD subjects compared to the other two groups (Figure 1). However, the CD4^+^/CD8^+ ^ratio was significantly lower in COPD subjects (0.70) compared to smokers (1.4; p < 0.001) and HNS (1.0; p < 0.001). There was also a significant increase in the proportion of B lymphocytes in COPD patients compared to smokers and HNS (Figure 1).

**Figure 2:**
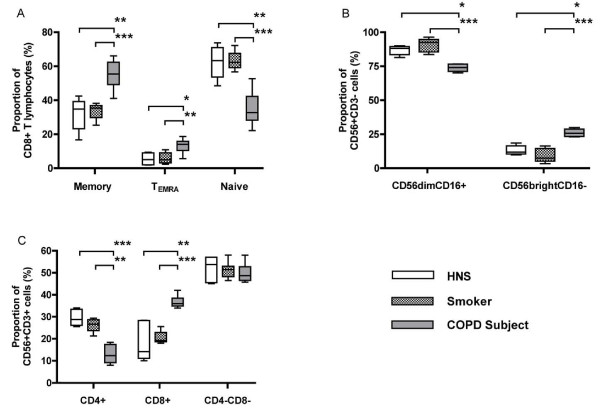
**Proportion of CD8+ T lymphocyte subsets (Panel A), NK (CD56^+^CD3^-^) subsets (Panel B) and NKT-like (CD56^+^CD3^+^) subsets (Panel C) from the induced sputum of HNS (n = 5), smokers (n = 10) and COPD subjects (n = 11)**. Panel A shows the proportion of highly cytotoxic effector memory cells (T_EMRA_; CD8^+^CD45RO^+^RA^+^CD62L^-^) was significantly increased in COPD subjects compared to HNS (*: p < 0.05) and smokers (**: p < 0.01). Panel B shows the proportion of CD56^bright^CD16^- ^NK cells was significantly increased in COPD subjects compared to HNS (*: p < 0.05) and smokers (***: p < 0.001). Panel C shows significantly more CD8^+^CD56^+^CD3^+ ^cells in the induced sputum of COPD subjects compared to HNS (**: p < 0.01) and smokers (***: p < 0.001).

Further analysis of the CD8^+ ^T lymphocytes by flow cytometry revealed that COPD subjects had an increased proportion of memory cells (CD45RO^+^RA^-^) and a reduction in the proportion of naïve cells (CD45RO^-^RA^+^) compared to the other two groups (Figure 2A). The proportion of T_EMRA _cells (CD45RO^+^RA^+^) was also higher in COPD subjects.

The subpopulation split of NK cells showed a higher proportion of immunoregulatory CD56^bright^CD16^- ^NK cells in COPD subjects, compared to the other two groups (Figure 2B ). There was no measurable difference in the proportion of NK cells expressing CD8 in any of the three groups (data not shown).

NKT-like (CD56^+^CD3^+^) cells in COPD subjects showed an increased proportion expressing CD8 and a decreased proportion expressing CD4 (Figure 2C), compared to the other two groups. There was no difference between groups for the double negative fraction.

### Expression of cytotoxic effector molecules

**Figure 3:**
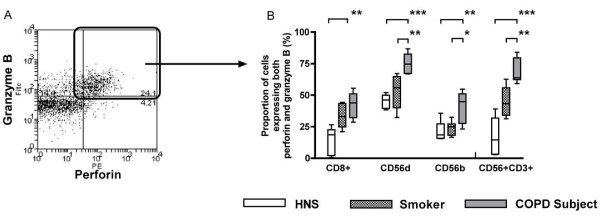
**Representative flow cytometry plot (Panel A) showing the expression of both granzyme B and perforin (Panel B) in CD8^+ ^T lymphocytes, CD56^dim^CD16^+ ^NK cells, CD56^bright^CD16^- ^NK cells and NKT-like (CD56^+^CD3^+^) cells from HNS (n = 5), smokers (n = 10) and COPD subjects (n = 11)**. Double stained cells (Panel B) are deemed cytotoxic. *: p < 0.05, **: p < 0.01, ***: p < 0.001.

The expression of perforin and granzyme B were studied in CD8^+ ^T lymphocytes, CD56^dim^CD16^+ ^NK cells, CD56^bright^CD16^- ^NK cells and NKT-like (CD56^+^CD3^+^) cells (Figure 3).

The proportion of CD8^+ ^T lymphocytes expressing both perforin and granzyme B was significantly higher in COPD subjects (43.9) compared to HNS (18.6; p < 0.01) (Figure 3B). The proportions of both CD56^dim^CD16^+ ^and CD56^bright^CD16^- ^NK cells expressing both perforin and granzyme B were also significantly higher in COPD subjects (74.6 and 44.8, respectively) compared to both HNS (46.2; p < 0.001 and 18.5; p < 0.01) and smokers (55.9; p < 0.01 and 24.9; p < 0.05)(Figure 3B). The proportion NKT-like (CD56^+^CD3^+^) cells expressing both perforin and granzyme B were also significantly higher in COPD subjects (63.8) compared to both HNS (14.5; p < 0.001) and smokers (43.4; p < 0.01)(Figure 3B).

The proportion of CD8^+ ^T lymphocytes that expressed only perforin and no granzyme B was significantly higher in both smokers (8.0; p < 0.01) and COPD subjects (4.6; p < 0.05) than in HNS (1.4) (data not shown). The proportion of NKT-like (CD56^+^CD3^+^) cells that expressed only perforin and no granzyme B was significantly higher in both smokers (14.0; p < 0.001) and HNS (13.8; p < 0.01) than in COPD subjects (5.4) (data not shown). There was no difference in the proportions of CD56^dim^CD16^+ ^NK cells and CD56^bright^CD16^- ^NK cells expressing only perforin and no granzyme B. Examining the proportion of cells expressing only granzyme B and no perforin revealed no differences between groups and cell types (data not shown).

### Cytotoxic activity of CD56^+ ^cells

To establish if the increased proportions of CD56^+ ^cells that expressed both perforin and granzyme B were cytotoxic in the LDH release assay, CD56^+ ^cells were immunomagnetically purified from induced sputum. All samples were ≥ 90% pure with respect to B lymphocytes, helper T lymphocytes, CD8^+ ^T lymphocytes, neutrophils and monocytes (Table 4).

**Table 4:** Purity of immunomagnetically separated CD56^+ ^cells from the induced sputum of the studied groups.

	HNS	Smokers	COPD subjects
**CD56^+ ^cells**	91.7 (± 0.8)	94.3 (± 1.1)	94.8 (± 1.4)
**B cells (CD19^+^)**	2.2 (± 0.4)	1.8 (± 0.8)	1.4 (± 0.7)
**Helper T cells (CD4^+^)**	1.4 (± 0.5)	0.7 (± 0.1)	0.6 (± 0.5)
**Cytotoxic T cells (CD8^+^)**	0.9 (± 0.6)	1.9 (± 0.6)	0.9 (± 0.4)
**Neutrophils (CD16^+^)**	1.1 (± 0.7)	0.9 (± 1.5)	0.4 (± 0.7)
**Macrophages (CD14^+^)**	1.7 (± 0.9)	1.3 (± 0.7)	0.9 (± 1.6)

Results are expressed as mean with standard deviation in brackets.

**Figure 4:**
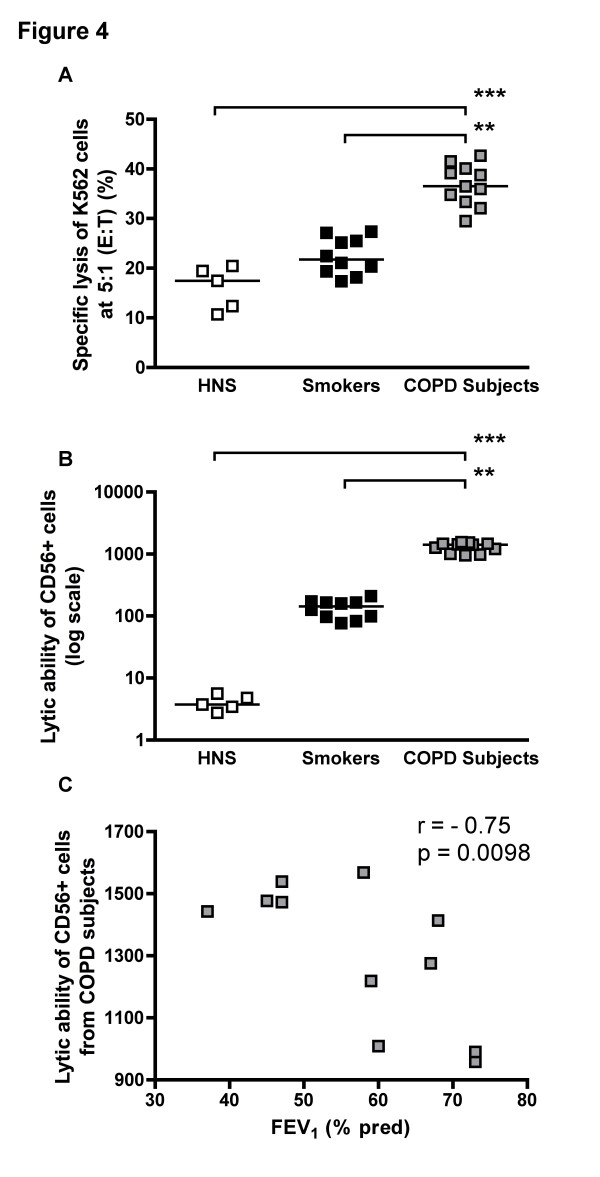
**Cytotoxic activity of CD56^+ ^cells (Panel A), biological lytic activity (Panel B) and correlation of cytotoxic activity of CD56^+ ^cells and lung function in COPD subjects (Panel C)**. Immunomagnetically separated CD56^+ ^cells were significantly more cytotoxic in COPD subjects than in HNS (***: p < 0.001) and smokers (**: p < 0.01) and were inversely correlated with FEV_1_.

Using the same number of effector cells (effector to target ratio of 5:1) the CD56^+ ^cells from COPD subjects were significantly more cytotoxic (36.8% specific lysis) than those from smokers (22.4%; p < 0.01) and HNS (16.1%; p < 0.001) (Figure 4A). When taking into account for the differences in cell numbers on overall cytotoxicity, by examining the product of CD56 cell numbers and lytic activity of the CD56^+ ^cells from COPD subjects (which we have termed biological lytic activity) the significant differences remain (Figure 4B) and this inversely correlated with lung function, as assessed by FEV_1 _measurement (r = -0.75; p = 0.0098) (Figure 4C).

### Adhesion molecule expression

**Figure 5:**
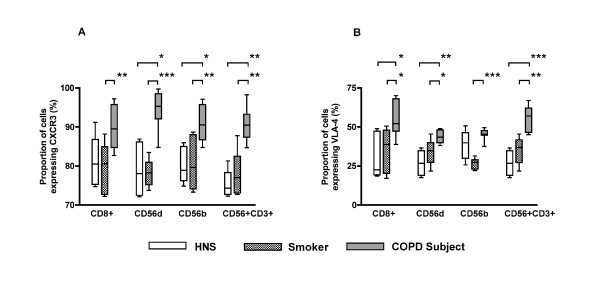
**Expression of CXCR3 (Panel A) and VLA-4 (Panel B) in CD8^+ ^T lymphocytes, CD56^dim^CD16^+ ^NK cells, CD56^bright^CD16^- ^NK cells and NKT-like (CD56^+^CD3^+^) cells from HNS (n = 5), smokers (n = 10) and COPD subjects (n = 11)**. CXCR3 expression was significantly higher in COPD subjects across all cell type compared to smokers. VLA-4 expression was also higher. *: p < 0.05, **: p < 0.01, ***: p < 0.001.

To examine a possible mechanism of selected cell recruitment to the lung the adhesion molecules CXCR3 and VLA-4 were measured. The proportion of CD8+ T lymphocytes, CD56^dim^CD16^+ ^NK cells, CD56^bright^CD16^- ^NK cells and NKT-like cells expressing CXCR3 was significantly higher in COPD subjects than in smokers (Figure 5A). The proportions of these cells expressing VLA-4 were also significantly higher in COPD subjects than in smokers (Figure 5B).

## Discussion

Here we report for the first time that NK cells (CD56^+^CD3^-^) and NKT-like cells (CD56^+^CD3^+^) from the induced sputum of COPD subjects are increased in both number and proportion and have a higher cytotoxic ability compared to HNS and smokers.

In this study we have also confirmed the findings of others, namely predominant neutrophilia [14], the presence of significantly more lymphocytes in COPD subjects, and a reduced CD4/CD8 ratio [15]. The increased proportion of B lymphocytes in the induced sputum of COPD subjects compared to smokers has not previously been reported, although B cell follicles have been demonstrated previously in both the small airways of COPD subjects [16,17] and the lung parenchyma [18] highlighting a potentially important role for these cells in COPD.

The proportion of highly cytotoxic T_EMRA _cells within the CD8^+ ^fraction was significantly increased in COPD subjects compared to smokers and HNS. This increased proportion of T_EMRA _cells, which are deemed highly cytotoxic due to their high perforin content [19], has not been previously reported in induced sputum and it is possible to hypothesise that these cells are causing some of the damage seen in the COPD airways. Furthermore, memory cells (CD45RO^+^RA^-^) within the CD8^+ ^fraction were also significantly increased in COPD subjects with a corresponding decrease in naïve cells (CD45RO^-^RA^+^). These memory cells could accelerate the inflammatory response seen in the COPD lung through rapid activation and secretion of mediators.

The proportion of NK cells was significantly increased in induced sputum in COPD subjects compared to smokers and HNS. Human NK cells can be divided into two subsets, namely CD56^dim^CD16^+ ^and CD56^bright^CD16^-^. In inflammatory lesions CD56^bright^CD16^- ^NK cells predominate and are thought to play an immunoregulatory role [20]. In the COPD subjects we also show a significantly increased proportion of the CD56^bright^CD16^- ^NK cells compared to smokers and HNS, suggesting a possible regulatory role for these cells in the inflammatory environment of the COPD lung.

The overall proportion of NKT-like cells was increased in COPD subjects. The proportion of these cells that expressed CD8 was significantly increased with a corresponding decrease in those expressing CD4. The double negative CD4^-^CD8^- ^NKT cells were not different between the groups. These CD8^+ ^NKT cells are known to produce predominantly Th1-type cytokines [21-24], which could influence the cytokine milieu within the lung to one which is pro-inflammatory. Interestingly, no difference in cytotoxic ability of the three NKT-like subsets has been reported to date.

As well as showing an increased number of CD8^+ ^T lymphocytes, NK cells and NKT-like cells, we have also shown that a significantly higher proportion of *all *these cell types express *both *intracellular perforin *and *granzyme B in COPD subjects compared to the other two groups. Perforin is an important mediator of granzyme B effector function in that it facilitates granzyme B access to the target cell. Hence the presence of both mediators is crucial for cell killing and our findings highlight the potential importance of these killer cells in the pathogenesis of COPD. Release of perforin and granzyme B by CD8^+ ^T lymphocytes is hypothesised to be an important mechanism in the development of COPD [25].

The increased proportion of all cell types expressing both mediators in COPD subjects leads one to hypothesise that these cells are therefore more cytotoxic and thus able to cause some of the damage seen in the COPD lung. To confirm this hypothesis CD56^+ ^cell cytotoxicity was measured using the LDH-release assay. Here we show, for the first time, that CD56^+ ^cells have greater specific lysis in COPD subjects than HNS and smokers. However, biological lytic activity depends on both the number of cells and specific lysis of each cell. The product of the numbers and specific lysis is greater in COPD subjects than HNS and smokers and is negatively correlated with FEV_1 _in COPD subjects.

Both the increased proportion and cytotoxicity of NK (CD56^+^CD3^-^) and NKT-like (CD56^+^CD3^+^) cells present in the induced sputum of smokers with COPD is contrary to what was observed in the peripheral blood of the same group [12]. This raises the possibility that the cells are being selectively recruited to the lung. The increased proportion of cells expressing CXCR3 and VLA-4 support this hypothesis. Previously it has been reported that the number of CXCR3^+ ^cells in the epithelium and submucosa of COPD subjects are increased [26] and correspondingly CXCR3-chemokines are also increased in sputum from COPD subjects [27].

The present study has some limitations that deserve comment. Firstly, seven patients received inhaled corticosteroids. For this reason, the results obtained in patients receiving or not receiving inhaled corticosteroids were compared, and no significant differences were found. Secondly, results generated from induced sputum will reflect changes in the upper airways but will not necessarily reflect lower airway changes or differences within bronchial tissue. However, it is a convenient and non-invasive research tool and differences found in induced sputum need to be pursued [28,29]. It would be important to follow up our findings by investigating bronchoalveolar lavage and bronchial biopsies in these groups of patients.

Of note is that the COPD subjects included current and ex-smokers and that there was no difference in terms of NK and NKT-like cell numbers or cytotoxicity between the two groups. A number of authors have suggested that the airway inflammatory process persists in ex-smokers with COPD [8,9,30]. Indeed, a pooled analysis study demonstrated that there is no significant difference in the inflammatory cell types and markers between smokers and ex-smokers with established COPD [31]. Alternatively, it could be that the changes we have described are a consequence of airway damage and therefore reflect previous smoking induced damage of the airways. It is not possible with our current data to determine whether these changes are a consequence or part of the mechanism of airway limitation.

In accordance with others [11,15,32,33] we have shown that sputum cells treated with DTT are suitable for flow cytometric analysis of both extracellular markers and intracellular proteins in lymphocytes. We have also shown that DTT-treated cells are suitable for use with a cytotoxicity assay.

## Conclusion

In summary, we have shown an increased proportion of three types of cytotoxic cells, namely CD8^+ ^T lymphocytes, NK and NKT-like cells, in the induced sputum of COPD subjects and have demonstrated for the first time that NK and NKT cells are significantly more cytotoxic in COPD subjects than smokers and HNS. This complements our previous finding of reduced numbers and cytotoxicity of these cells in the periphery. These cells may play a part in the pathogenesis of disease or may be a consequence of the underlying lung pathology. Further studies are needed to explore possible mechanisms by which CD8^+ ^T lymphocytes, NK and NKT-like cells may contibute to disease pathogenesis.

## Competing interests

The authors declare that they have no competing interests.

## Authors' contributions

RAU carried out the experimental work and wrote the manuscript. JRL and IT participated in the study's design and edited the manuscript. LF and JC conceived the study, participated in its' design and co-ordination, and edited the manuscript. All authors read and approved the final manuscript.
